# Examining Differential Resilience Mechanisms by Comparing ‘Tipping Points’ of the Effects of Neighborhood Conditions on Anxiety by Race/Ethnicity

**DOI:** 10.3390/healthcare6010018

**Published:** 2018-02-20

**Authors:** Emil Nicolae Coman, Helen Zhao Wu

**Affiliations:** 1 Health Disparities Institute, University of Connecticut Health Center, Hartford, CT, USA; 2 Department of Psychiatry, University of Connecticut Health Center, Hartford, CT, USA; zwu@uchc.edu

**Keywords:** health disparities, resilience, stress, heterogeneity of effects

## Abstract

Exposure to adverse environmental and social conditions affects physical and mental health through complex mechanisms. Different racial/ethnic (R/E) groups may be more or less vulnerable to the same conditions, and the resilience mechanisms that can protect them likely operate differently in each population. We investigate how adverse neighborhood conditions (neighborhood disorder, NDis) differentially impact mental health (anxiety, Anx) in a sample of white and Black (African American) young women from Southeast Texas, USA. We illustrate a simple yet underutilized segmented regression model where linearity is relaxed to allow for a shift in the strength of the effect with the levels of the predictor. We compare how these effects change within R/E groups with the level of the predictor, but also how the “tipping points,” where the effects change in strength, may differ by R/E. We find with classic linear regression that neighborhood disorder adversely affects Black women’s anxiety, while in white women the effect seems negligible. Segmented regressions show that the Ndis → Anx effects in both groups of women appear to shift at similar levels, about one-fifth of a standard deviation below the mean of NDis, but the effect for Black women appears to start out as negative, then shifts in sign, i.e., to increase anxiety, while for white women, the opposite pattern emerges. Our findings can aid in devising better strategies for reducing health disparities that take into account different coping or resilience mechanisms operating differentially at distinct levels of adversity. We recommend that researchers investigate when adversity becomes exceedingly harmful and whether this happens differentially in distinct populations, so that intervention policies can be planned to reverse conditions that are more amenable to change, in effect pushing back the overall social risk factors below such tipping points.

## 1. Introduction

The environment clearly affects our health [1], both physical and mental, and residents living in economically challenged areas experience worse health outcomes [2]. Some groups are affected more disproportionately, such as racial/ethnic (R/E) minorities in the United States [3,4]. Neighborhood conditions affect well-being and health in general and mental health in particular [5], primarily indirectly, through intermediaries like sleep quality [6,7], individual SES, perceptions of neighborhood quality, and psychosocial status [8], or the dysregulation of stress-related biological pathways, such as cortisol secretion [9]. Moreover, the effects of neighborhood adversity complement, and may intensify, the effects of personal stressors on psychological stress [10].

Neighborhood conditions can also have lifelong consequences [11], primarily through intermediate outcomes (such as educational attainment [12,13]). These disparate effects can accumulate over the course of one’s life [14] and therefore contribute to increasing health disparity effects [15]. While neighborhood residents rely on sources of resilience [16] or self-regulation [17] to protect themselves, they resort to such support resources differentially, i.e., to different degrees. There is evidence for both differential vulnerability to the same adverse conditions or hardship and a diminishing returns effect, which means fewer benefits from the same protective factors, such as higher income [18] or social contacts [19], for some minority R/E groups. The differential vulnerability-benefits theoretical approach to health disparities invites research questions such as whether the strength of the effect of a social stressor, like neighborhood distress or disorder (NDis), on health outcomes changes when neighborhood distress becomes excessive, overcoming the available resources of resilience to adversity; in other words, whether the effect may change in strength along the continuum of the predictor. 

Literature on resilience defines it as a “bouncing back” dynamic mechanism [20] that can be characterized by differential tipping points where such mechanisms start operating in full swing [21]. These points of effect-shifting can also differ by population. A simple way to detect such differential responses to adverse conditions is to step away from the simple linear effect assumption, which is still common in many analytical methods, and investigate instead *if and when* the effects of adverse conditions seem to change in how strongly they impact health outcomes. Such knowledge would provide valuable insight for interventions focused on “diversion” approaches, which are meant to divert away from risk, i.e. to try to reduce risk factors below the point where risk turns excessively harmful. 

We therefore examine the following research questions: Does neighborhood disorder impact health differentially in Black and white young women, and are the levels of the neighborhood adverse conditions, where the effect on anxiety changes in strength, R/E-specific? 

## 2. Methods 

### 2.1. Study Description 

The data comes from a larger longitudinal study on stress and substance use in young women, which was conducted between November 2006 and January 2012 in Southeast Texas [22,23]. Participants were selected from patients attending 1 of 6 University of Texas Medical Branch (UTMB) community-based family planning clinics. These clinics serve primarily low-income women with average annual income below $6000. The study was approved by the UTMB Institutional Review Board. For this analysis, we focused on a subset of data on only Black (n_B_ = 63) and white (n_W_ = 42) young women (excluding Hispanics), with complete values for predictor (only measured at baseline in the original study) and outcome.

### 2.2. Measures 

Anxiety was measured using 5 items from Carver’s 7-item BIS scale [24] that loaded on a distinct BIS factor according to a factor analysis in the full sample. BIS items were: “I feel worried because: I think someone is angry at me, *or* I think I’ve done poorly at something, *or* about making mistakes”; “criticisms hurts me”; and “I think something unpleasant is going to happen to me.” The BIS was also suggested in [25] to contain fewer than its original 7 items; its internal reliability Cronbach’s alpha was 0.696 in our initial sample. 

Neighborhood disorder (NDis) was measured using 10 items taken from the initial 14 individual-level neighborhood condition questions that measure both cohesion and disorder [26]. The NDis assesses how residents perceive problems related to safety and signs of physical neglect in their neighborhood (e.g., unsafe traffic and walking conditions, poor public transportation and broken curbs, vandalism; all were rated from “never” to “often”). The full list of questions can be found in Table 1 in [27]. Responses to each question were z-scored and averaged across questions to create individual-level summary measures of perceived neighborhood disorder. Cronbach’s alpha was found to be above 0.80 in [27] and 0.91 in [26], and was 0.83 in our initial full sample. Of note, such measures of stressful conditions, if merely adding up the total count of *presence* of problems or stressors (yes vs. no), are thought to form a different measurement structure, that of an index (formative measure) rather than a scale (reflective indicators measure) [28,29,30].

### 2.3. Analysis 

We tested R/E descriptive differences using chi-squared and t-tests (see Table 1). The main analysis simply tried to identify two distinct areas in the *outcome* (*predictor*) scatter plot, for which two linear slopes are estimated (e.g., going up, then down) instead of one, as in classical linear regression. Linear regressions of the BIS anxiety (Anx) outcome on neighborhood disorder (NDis) and on covariates that ensure better comparability of health outcomes across racial/ethnic groups (age, income, education, marital status, employment status) were tested in both groups simultaneously (with Stata’s multigroup *sem* command), then segmented (or piecewise) regressions were tested in each R/E group [31], as implemented in Stata’s [32] *nl hockey* command [33]. This regression approach simply relaxes the assumption that the effect of the predictor on the outcome is the same across all cases, such that two linear slopes are estimated instead of one. Hence the slope line is allowed to shift, or turn, and it does so at a particular level of the predictor, a value that is estimated and provided in the output as the tipping point (or breakpoint). We provide all software code and output online at http://bit.ly/disparitiestippingpoint (supplementary materials).

This shifting slope model is similar to latent growth modeling’s piecewise (or spline) option [34], in which two distinct linear time-predicted slopes meet in a “knot point”; an intuitive example is shown in the *Significance* magazine [35]. Such models presume a linear growth rate up to a time point, and then a different such slope afterward [36,37], as well as a turning time point. The segmented regression method instead can be applied to any Y(X) pair of variables (effect-cause), i.e., the predictor does not have to be time. 

## 3. Results

The two racial/ethnic (R/E) groups of young women differed only in terms of marital status (see Table 1), with more Black women reporting not being married and having a boyfriend and more white women reporting a cohabitating status. Notably, even though Black women reported significantly higher average neighborhood disorder levels ($\bar{\mathrm{NDis}}$_B_ = 0.18 vs. $\bar{\mathrm{NDis}}$_W_ = –0.18 for white women), their average level of BIS anxiety ($\bar{\mathrm{Anx}}$_B_ = 13.48) was significantly lower on average than that of their white counterparts ($\bar{\mathrm{Anx}}$_W_ = 14.57), which is a first potential indication of an effect previously called “flourishing despite adversity” [38], possibly linked to differential resilience (or coping) mechanisms. Lower incidence of psychological distress in Blacks than whites has been reported before [39], but the symptoms can be more severe and disabling in Blacks, for example when it comes to depression [40].

The overall (global) Ndis → Anx linear effects of neighborhood disorder on BIS anxiety differed slightly in the two groups (see Table 2), with standardized slope for Black women β_B_ = +0.189 (SE = 0.118, *p* = 0.109), net of covariates, vs. β_W_ = –0.020 (SE = 0.118, *p* = 0.161) for white women; the difference in βs, however, was not significant according to a Wald test: χ^2^(1) = 0.372, *p* = 0.542; or directly as a test of the difference between unstandardized βs: ∆_Bvs.W_β = 0.502 (SE = 0.823, z = 0.610, *p* = 0.542, following [41]). 

**Figure 1:**
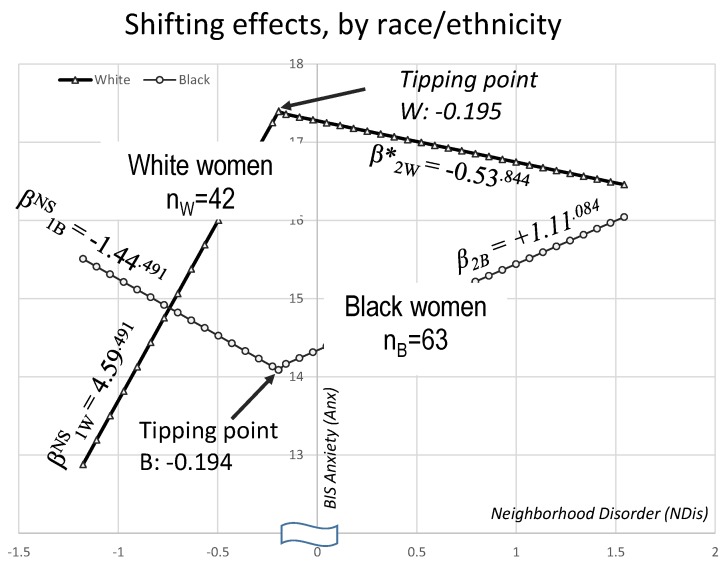
Linear slopes for neighborhood disorder → anxiety effects, shifting around similar tipping points.

When we tested models where two (instead of one) linear slopes are allowed and estimated in each group, the Ndis → Anx effects differed slightly by R/E, with the two tipping points quite similar, however. Table 2 and Figure 1 show that the Ndis → Anx effect in white women starts out positive, i.e., increasing Anx levels with higher NDis, then shifts to negative, i.e., lowering Anx, although technically both slopes are not significantly different from zero (as is their). In contrast, the Ndis → Anx effect starts out positive, then shifts to become negative and nearly significant, β_2B_ = +1.11 (SE = 0.61, *p* = 0.084), net of covariates (see Figure 1). The two R/E before-tipping-point slopes were not different [41]: ∆_B vs. W_β_1_ = 6.03, SE = 3.781, z = 1.595, *p* = 0.111, neither were the two after-the-tipping-point slopes, ∆_B vs. W_β_2_ = 1.64 (SE = 2.719, z = 0.603, *p* = 0.547).

To summarize, the level of neighborhood disorder at which its effect on anxiety changes in size is similar in the two groups. While the Black group does not show a significant effect of neighborhood disorder on anxiety below the tipping-point value, neighborhood disorder does have a detrimental effect on anxiety above this threshold. 

## 4. Discussion

We found that more nuanced health disparity (HD) conclusions can be reached by investigating differential effects on health by racial/ethnic (R/E) groups using a segmented (piecewise) regression method that allows the linear effects to switch/change. In particular, we found that the effect of neighborhood disorder, a measure of adverse environmental conditions, on anxiety operates differently in white than in Black young women, confirming prior findings of differential associations between neighborhood conditions in different R/E groups [39]. It appears that Black and white women handle challenging neighborhood conditions differently, considering how such conditions impact their mental health, anxiety in particular. We note that at about one-fifth of a standard deviation in neighborhood disorder below its mean, something happens that changes the dynamic of the *neighborhood disorder* → *anxiety* relationship in both white and Black young women, although the change is markedly different in the two groups.

Our findings add nuance to prior reports of how stressed neighborhoods contribute to psychological distress, such as depression [42], due to the social stress of neighborhoods with higher levels of deprivation [5]. The analysis reported here seems to indicate that Black women can better handle low to medium neighborhood disorder levels, but above a threshold (−0.195, on a z-score scale), these conditions start affecting their mental health more adversely than white women’s. This adds to the body of evidence for differential vulnerability to equal hardship, and for unequal gain of equal resources [43]. Our results provide a means of pinpointing at what level of stressors the differential vulnerability/gains effect changes in strength. While not significant except for the neighborhood → anxiety effect at higher neighborhood hardship levels for Black women, the patterns of shifting effects seen in Figure 1 suggest that Black women can better handle lower levels of distress due to neighborhood conditions, but above a certain threshold, the detrimental effects on mental health become more severe than that experienced by white women. 

This specific pattern supports differential vulnerability: a differential losses description of effects, where in our case the losses in terms of worse mental health due to neighborhood stressors differ by race/ethnicity. While it has been shown that gains in general are larger for “haves” than for “have-nots” [44], segmented/tipping point regression can nuance the differential effects of losses due to hardship, such that in our case the “have-nots” also lose more healthwise when the level of hardship exceeds a level beyond which it is not managed well anymore with available psychological or community coping resources. Knowing when such effects turn more detrimental can inform policies to intervene and push back the set of adverse conditions below the tipping point, often by intervening on social ills more amenable to change [44], such as transportation or safety perceptions. 

The finding that effects shift at a certain tipping point to become stronger in one group (and weaker in another) suggest potential tailored interventions to reduce health disparities that are race/ethnic culturally appropriate and take into account the tipping point where the effects become more harmful, because they exceed the ability of personal or group resilience resources to cope with such adversities. Stressors from a range of sources (personal, work, or family-related, etc.) have additive and multiplicative effects on health [10], hence knowing when a critical mass is reached invites interventions to push back the stressors below the tipping point, so that handling them while preserving some equilibrium [20] is more feasible. 

### Limitations and Extensions 

The actual mechanisms leading to psychological stress are not fully revealed by this method, because this approach does not explain the causal mechanisms behind the shift in effect size, like moderation of more intricate analyses can; it only reveals tipping point where the effect shifts. The mechanisms through which neighborhood problems affect well-being and health in general and mental health in particular cover a wide range of possible pathways; for instance, 120 items that can describe neighborhoods and can impact well-being were listed in [45], according to residents themselves, while another 100 attributes important for mental health were enumerated in [46], and the causal pathways leading to actual health disparities between these two sets of factors would spell out a large big-picture causal model, and hence would invite complex statistical modeling.

As a clear limitation, the small sample size invites larger replications of the processes explored here. The segmented/piecewise regression approach we illustrate allows for nuanced comparisons of distinct mechanisms differentially affecting health outcomes, within distinct racial/ethnic (or other) populations. It is, however, a simple first methodological step away from the common assumption of linearity of the effect of interest, hence it provides an initial view into the heterogeneity of effects [47], by splitting one linear effect into two linear effects. An even more nuanced analytical method that uses a stronger estimation algorithm and better handles sample sizes like our own (or even smaller [48]) is the mixture (i.e., latent class [49]) approach, which is a repositioning of classical analytical methods that customarily assume only one population behind the data. Mixture models can truly open the door to investigations of heterogeneity of effects or of population [50].

Instead of assuming a precise neighborhood tipping point where the effect shifts, one can investigate whether there exist in fact two (or more) subpopulations within each racial/ethnic group, differing in their average neighborhood distress (but with overlapping distributions), and across which the neighborhood disorder → anxiety effect also varies. In fact, any classic one-group analytic model (e.g., a regression) can be seen as a particular case of a corresponding latent class analysis (e.g., latent class regression) in which there is only one class, and that class is known (hence the *knownclass* option in Mplus [51]), or, in other words, only one population is assumed [49]. We illustrate this option in the appendix, where the *Neighborhood Disorder* → *Anxiety* regression is re-modeled as a one-latent class mixture regression, which should replicate the parameters from the classic regression for the same sample size and model. 

A two-class multi-group mixture analysis (or latent class model; see Mplus [51] input and output in the online appendix) confirmed our pattern of findings from the segmented regression, that the effects on anxiety vary across the two classes of white and Black women exposed to different levels of neighborhood distress. As an added benefit, the latent class (mixture) regression also relaxes the assumptions of the sharp separation of cases along the predictor values needed in the segmented regression, i.e., the tipping point is not a single value that splits the sample, but cases from each class overlap in their distribution of values, in both predictor and outcome. Therefore, instead of the two similar –0.19 tipping points (in neighborhood disorder z-scores) yielded by the segmented regression in the sample of white and Black sample women, the mixture analysis indicates two classes of women in each racial/ethnic group, with neighborhood disorder means of –0.25 and –0.62 (in the white group) and –0.28 and 0.61 (in the Black group).

Additionally, Mplus software can test the R/E differences in effects for significance in both latent classes; both differences were found to be significant within classes between white and Black women (see online appendix for details). Conveniently, the latent class approach can relax even the strict assumption that every person belongs to a racial/ethnic group with 100% certainty, which in the modern social landscape is clearly an exaggeration, because individuals identify along a continuum with several racial and cultural group identities [52]. Mplus allows some uncertainty (technically, measurement error) in the group (class) assignment [53], to be built in, which naturally will reduce the biases in estimates of health disparity effects. 

## 5. Conclusions

We found that neighborhood conditions affect differently mental health in two racial/ethnic groups of young women (white and Black), and that these effects themselves might shift when neighborhood conditions exceed a critical mass burden level. Our illustration was meant to mainly demonstrate the ease and meaningfulness of the segmented regression approach, and of extensions of this method. We note that both the shifting effects and the tipping point estimates depend heavily on the choice of covariates; here we chose to include all relevant socio-demographic variables as covariates, but covariates can be added more confidently after prior causal analysis of the processes at work [54]. We hope that our report will encourage further, more nuanced, interrogations of health data from multiple racial/ethnic groups.

## Figures and Tables

**Table 1:** Descriptives of demographic characteristics of the sample, and of predictor and outcome measures, by race/ethnic (R/E) groups.

Race/Ethnicity→ Measures↓	White (n_W_ = 42)		Black (n_B_ = 63)		
	*N*	*%s*	*N*	*%s*	*p_∆_*
*Employment*					0.089
Unemployed	14	34	25	40	
Homemaker	5	12	5	8	
Part-time	10	24	5	8	
Full-time	12	29	27	44	
*Education*					0.322
<High school	12	29	23	37	
High school	21	50	33	52	
>High school	9	21	7	11	
*Marital status*					0.002
Married	8	19	4	6	
Cohabitating	17	40	14	22	
With boyfriend	10	24	38	60	
No boyfriend	7	17	7	11	
*Mean (M) and SD*	*M*	*SD*	*M*	*SD*	
Age	21.90	3.36	22.46	3.71	0.219
Income ($US thousands/year)	4.39	7.50	5.27	6.96	0.306
Neighborhood disorder (NDis)	–0.18	0.56	0.18	0.65	0.008
BIS anxiety (Anx)	14.57	2.54	13.48	2.04	0.002

Notes: Bold numbers in the higher value group when *p* < 0.05; *p*_∆_ is the *p* values for R/E difference tests.

**Table 2:** Estimates of classic linear slopes and segmented effect estimates and tipping points, by race/ethnic (R/E) groups.

Race/Ethnicity→ Effects↓	White (n_W_ = 42)		Black (n_B_ = 63)	
Effects and tipping points	Estimate	SE	Estimate	SE
Classic NDis →anxiety effect	–0.02 ^NS^	(0.12)	0.19 ^A ^	(0.12)
NDis → anxiety effect 1	4.59 ^NS^	(3.17)	–1.44 ^NS^	(2.06)
NDis tipping point	–0.195 ^NS^	(0.19)	–0.194 *	(0.09)
NDis → anxiety effect 2	–0.53 ^NS^	(2.65)	*1.11 ^B^*	(0.61)

Notes: * and bold indicate *p* < 0.05; *italics: p* < 0.10; A: *p* = .0109; B: *p* = 0.084; SE: standard error; NS means statistically non-significant.

## Supplementary Materials

The following are available online at http://www.mdpi.com/2227-9032/6/1/18/s1. All analyses inputs/code and outputs in Stata and Mplus reported, plus graphical displays; also posted at http://bit.ly/disparitiestippingpoint.

Supplementary File 1
