# Supplementary material for: Examining Differential Resilience Mechanisms by Comparing ‘Tipping Points’ of the Effects of Neighborhood Conditions on Anxiety by Race/Ethnicity
Source: Healthcare (Basel). 2018 Feb 20;6(1):18. doi: 10.3390/healthcare6010018 (PMC5872225; doi:10.3390/healthcare6010018)
Supplement: Supplementary File 1 [file healthcare-06-00018-s001.docx]

Article

Examining Differential Resilience Mechanisms by Comparing ‘Tipping Points’ of the Effects of Neighborhood Conditions on Anxiety by Race/Ethnicity

Emil Nicolae Coman, Helen Zhao Wu

**Supplementary Materials**

*Examining differential resilience mechanisms by comparing ‘tipping points’ of the effects of neighborhood problems on anxiety by race/ethnicity*

**1. Examine Mean of Outcome in Stata:**

Variable | Type Obs Mean [95% Conf. Interval]

-------------+---------------------------------------------------------------

anx0 | Arithmetic 105 13.91429 13.46834 14.36023

| Geometric 105 13.70591 13.23483 14.19376

| Harmonic 105 13.467 12.94274 14.03551

-----------------------------------------------------------------------------

**2. Examine Means of Outcome and Key Predictor in Stata:**

. sum anx0 disomean

Variable | Obs Mean Std. Dev. Min Max

-------------+---------------------------------------------------------

anx0 | 105 13.91429 2.30432 6 20

disomean | 105 .0393986 .6377499 -1.177697 1.459673

|  | |  |
| --- | --- | --- |
|  | |  |
|  |  | |
|  |  | |

**3. Scatterplots by Groups**

|  |  |
| --- | --- |

*3.1. Compare Linear and Constant Effects First (in Each Group)*

sem (empl2 empl3 empl4 ed2 ed3 mar2 mar3 mar4 disomean -> anx0), group(blvswh) ginvariant(none) standardized

Endogenous variables

Observed: anx0

Exogenous variables

Observed: empl2 empl3 empl4 ed2 ed3 mar2 mar3 mar4 disomean

Fitting target model:

Iteration 0: log likelihood = -599.37603

Iteration 1: log likelihood = -599.37603

Structural equation model Number of obs = 105

Grouping variable = blvswh Number of groups = 2

Estimation method = ml

Log likelihood = -599.37603

Group : 0 White Number of obs = 42

------------------------------------------------------------------------------

| OIM

| Coef. Std. Err. z P>|z| [95% Conf. Interval]

-------------+----------------------------------------------------------------

Structural |

anx0 |

empl2 | .195019 .1598583 1.22 0.222 -.1182976 .5083355

empl3 | -.0795517 .1869104 -0.43 0.670 -.4458894 .2867859

empl4 | .1384325 .1788993 0.77 0.439 -.2122038 .4890688

ed2 | -.2553639 .1903742 -1.34 0.180 -.6284906 .1177627

ed3 | -.0365495 .2110858 -0.17 0.863 -.4502699 .377171

mar2 | -.1019209 .209593 -0.49 0.627 -.5127156 .3088738

mar3 | .0102134 .2269477 0.05 0.964 -.434596 .4550227

mar4 | -.1695903 .2078115 -0.82 0.414 -.5768934 .2377129

disomean | .0199661 .1616995 0.12 0.902 -.2969591 .3368913

_cons | 6.129079 .7941869 7.72 0.000 4.572501 7.685656

-------------+----------------------------------------------------------------

var(e.anx0)| .9004345 .0854719 .7475729 1.084553

------------------------------------------------------------------------------

Group : 1 Blacks Number of obs = 63

------------------------------------------------------------------------------

| OIM

| Coef. Std. Err. z P>|z| [95% Conf. Interval]

-------------+----------------------------------------------------------------

Structural |

anx0 |

empl2 | -.2997678 .1100918 -2.72 0.006 -.5155437 -.0839919

empl3 | .0511577 .114311 0.45 0.654 -.1728877 .2752032

empl4 | .0601482 .1224938 0.49 0.623 -.1799353 .3002317

ed2 | -.2954934 .1161345 -2.54 0.011 -.5231129 -.0678739

ed3 | .0057402 .1242149 0.05 0.963 -.2377164 .2491969

mar2 | -.2381305 .2078029 -1.15 0.252 -.6454167 .1691556

mar3 | -.2591216 .2249716 -1.15 0.249 -.7000578 .1818145

mar4 | .023049 .1747469 0.13 0.895 -.3194486 .3655467

disomean | .1885108 .1176123 1.60 0.109 -.042005 .4190266

_cons | 7.376558 .7200611 10.24 0.000 5.965264 8.787851

-------------+----------------------------------------------------------------

var(e.anx0)| .7303354 .0888882 .5753388 .9270882

------------------------------------------------------------------------------

LR test of model vs. saturated: chi2(0) = 0.00, Prob > chi2 = .

| Wald Test Score Test

| chi2 df p>chi2 chi2 df p>chi2

-------------+----------------------------------------------------------------

Structural |

anx0 |

empl2 | 5.964 1 0.0146 . . .

empl3 | 0.371 1 0.5424 . . .

empl4 | 0.218 1 0.6409 . . .

ed2 | 0.006 1 0.9387 . . .

ed3 | 0.029 1 0.8639 . . .

mar2 | 0.185 1 0.6672 . . .

mar3 | 0.479 1 0.4887 . . .

mar4 | 0.510 1 0.4751 . . .

disomean | 0.372 1 0.5417 . . .

*3.2. Mplus 1-Class Latent Class/Mixture Regression for White and Black Women (equivalent to 2-Group SEM Above)*

Usevariables = anx0 disomean age income

ed2 ed3 mar2 mar3 mar4 empl2 empl3 empl4;

CLASSES = BlWh (2) ;!Lupdown (2) 2nd MvsL (2)is observed 'class'

KNOWNCLASS = BlWh (blvswh = 0 blvswh = 1);

ANALYSIS: TYPE = MIXTURE;

Model:

%overall%

anx0 on disomean age income ed2 ed3 mar2 mar3 mar4 empl2 empl3 empl4 ;

!~~~~~~~~~~~~~~~~~~two sections 1 for white and Black groups

%BlWh#1% ! Model white:

anx0 on disomean (WL1beta)

age income ed2 ed3 mar2 mar3 mar4 empl2 empl3 empl4 ;

disomean (WL1AveX);

%BlWh#2% !.Lupdown#1 Model Black:

anx0 on disomean (BL1beta)

age income ed2 ed3 mar2 mar3 mar4 empl2 empl3 empl4 ;

disomean (BL1AveX);

Model constraint:

NEW( dL1bBvsW );

dL1bBvsW = BL1beta-WL1beta; !~OUTPUT BELOW

FINAL CLASS COUNTS AND PROPORTIONS FOR THE LATENT CLASSES

BASED ON THE ESTIMATED MODEL

Latent Classes ! Actual white and Black observed groups defined above as KNOWNLASS

1 33.00000 0.47143 !N lower than 42 in Stata above

2 37.00000 0.52857 !N lower than 63 in Stata above

MODEL RESULTS

Two-Tailed

Estimate S.E. Est./S.E. P-Value

Latent Class 1 (0) !white women

ANX0 ON

DISOMEAN 0.188 1.197 0.157 0.875

AGE -0.037 0.151 -0.243 0.808

INCOME 0.092 0.060 1.534 0.125

ED2 -1.954 0.993 -1.968 0.049

ED3 -0.169 1.808 -0.094 0.925

MAR2 -0.215 1.735 -0.124 0.901

MAR3 0.072 1.970 0.037 0.971

MAR4 -0.459 1.121 -0.409 0.682

EMPL2 1.032 1.941 0.532 0.595

EMPL3 -1.703 1.107 -1.539 0.124

EMPL4 -0.111 0.765 -0.145 0.884

Latent Class 2 (1) !Black women

ANX0 ON

DISOMEAN 1.112 0.427 2.604 0.009

AGE 0.042 0.090 0.471 0.638

INCOME 0.068 0.035 1.946 0.052

ED2 -1.231 0.600 -2.052 0.040

ED3 1.221 0.672 1.818 0.069

MAR2 -1.593 1.068 -1.492 0.136

MAR3 -2.727 0.843 -3.233 0.001

MAR4 -2.151 1.086 -1.982 0.048

EMPL2 0.456 0.846 0.539 0.590

EMPL3 1.919 0.964 1.992 0.046

EMPL4 -0.649 0.695 -0.934 0.350

New/Additional Parameters !difference in slopes (Black-white)

DL1BBVSW 0.924 1.271 0.727 0.467

**4. Segmented/‘Hockey Stick’ Regression in Entire Sample (ALL)**

. nl hockey anx0 disomean

(obs = 105)

Iteration 0: residual SS = 551.2757

…

Iteration 5: residual SS = 547.5378

Source | SS df MS Number of obs = 105

-------------+------------------------------ F( 3, 101) = 0.29

Model | 4.69075914 3 1.56358638 Prob > F = 0.8337

Residual | 547.537812 101 5.42116646 R-squared = 0.0085

-------------+------------------------------ Adj R-squared = -0.0210

Total | 552.228571 104 5.30989011 Root MSE = 2.32834

Res. dev. = 471.3816

(hockey)

------------------------------------------------------------------------------

anx0 | Coef. Std. Err. t P>|t| [95% Conf. Interval]

-------------+----------------------------------------------------------------

breakpoint | -.5109591 2.449796 -0.21 0.835 -5.370696 4.348778

slope_l | -.32657 2.37602 -0.14 0.891 -5.039955 4.386815

slope_r | -.0827664 .3740211 -0.22 0.825 -.8247237 .6591909

cons | 13.62644 1.938869 7.03 0.000 9.780242 17.47263

------------------------------------------------------------------------------

* Parameter cons taken as constant term in model & ANOVA table

(SEs, P values, CIs, and correlations are asymptotic approximations)

**5. Segmented/‘Hockey Stick’ Regression in White Women**

. nl hockey anx0 disomean if blvswh ==0

(obs = 42)

Iteration 0: residual SS = 290.2261

Iteration 1: residual SS = 267.3509

Iteration 2: residual SS = 267.3373

Iteration 3: residual SS = 267.3157

Iteration 4: residual SS = 266.5245

Iteration 5: residual SS = 264.2298

Source | SS df MS Number of obs = 42

-------------+------------------------------ F( 1, 40) = 0.01

Model | .055949003 1 .055949003 Prob > F = 0.9271

Residual | 264.229765 40 6.60574413 R-squared = 0.0002

-------------+------------------------------ Adj R-squared = -0.0248

Total | 264.285714 41 6.44599303 Root MSE = 2.570164

Res. dev. = 196.4351

(hockey)

------------------------------------------------------------------------------

anx0 | Coef. Std. Err. t P>|t| [95% Conf. Interval]

-------------+----------------------------------------------------------------

breakpoint | 1.947078 . . . . .

slope_l | -.0657889 .714854 -0.09 0.927 -1.510563 1.378985

slope_r | .0954347 . . . . .

cons | 14.55978 .4162951 34.97 0.000 13.71842 15.40114

**6. Segmented/‘Hockey Stick’ Regression in White Women — With Covariates**

. nl hockey anx0 disomean age income empl2 empl3 empl4 ed2 ed3 mar2 mar3 mar4 if blvswh ==0

(obs = 33)

Iteration 0: residual SS = 28704.22

Iteration 18: residual SS = 168.3065

Source | SS df MS Number of obs = 33

-------------+------------------------------ F( 13, 19) = 0.57

Model | 65.7541762 13 5.05801355 Prob > F = 0.8477

Residual | 168.30643 19 8.85823315 R-squared = 0.2809

-------------+------------------------------ Adj R-squared = -0.2111

Total | 234.060606 32 7.31439394 Root MSE = 2.976278

Res. dev. = 147.4161

(hockey)

------------------------------------------------------------------------------

anx0 | Coef. Std. Err. t P>|t| [95% Conf. Interval]

-------------+----------------------------------------------------------------

breakpoint | -.1947891 .1865727 -1.04 0.310 -.5852904 .1957121

slope_l | 4.592469 3.170316 1.45 0.164 -2.043078 11.22802

slope_r | -.5303888 2.654296 -0.20 0.844 -6.085894 5.025116

age | .0566192 .2334242 0.24 0.811 -.4319433 .5451817

income | .0825214 .0820143 1.01 0.327 -.0891365 .2541793

empl2 | .5108886 1.888595 0.27 0.790 -3.441986 4.463764

empl3 | -2.590196 1.79269 -1.44 0.165 -6.34234 1.161947

empl4 | -.3915222 1.608897 -0.24 0.810 -3.758982 2.975938

ed2 | -2.419149 1.659865 -1.46 0.161 -5.893286 1.054989

ed3 | -1.963423 2.634753 -0.75 0.465 -7.478024 3.551179

mar2 | -.6448011 1.806821 -0.36 0.725 -4.426521 3.136919

mar3 | .0917912 2.287409 0.04 0.968 -4.695811 4.879393

mar4 | -.8047847 2.14825 -0.37 0.712 -5.301125 3.691555

cons | 18.28786 5.432111 3.37 0.003 6.918317 29.6574

------------------------------------------------------------------------------

**7. Segmented/‘Hockey Stick’ Regression in Black Women**

. nl hockey anx0 disomean if blvswh ==1

(obs = 63)

Iteration 0: residual SS = 261.0674

Iteration 1: residual SS = 258.4402

Iteration 2: residual SS = 233.857

Source | SS df MS Number of obs = 63

-------------+------------------------------ F( 2, 60) = 3.06

Model | 23.8572896 2 11.9286448 Prob > F = 0.0542

Residual | 233.856996 60 3.8976166 R-squared = 0.0926

-------------+------------------------------ Adj R-squared = 0.0623

Total | 257.714286 62 4.15668203 Root MSE = 1.974238

Res. dev. = 261.4155

(hockey)

------------------------------------------------------------------------------

anx0 | Coef. Std. Err. t P>|t| [95% Conf. Interval]

-------------+----------------------------------------------------------------

breakpoint | -1.079458 1.154914 -0.93 0.354 -3.389631 1.230715

slope_l | 2.173687 1.185362 1.83 0.072 -.1973903 4.544765

slope_r | .9870689 .4184036 2.36 0.022 .1501371 1.824001

cons | 17.05994 . . . . .

------------------------------------------------------------------------------* Parameter cons taken as constant term in model & ANOVA table

(SEs, P values, CIs, and correlations are asymptotic approximations)

**8. Segmented/‘Hockey stick’ regression in Black women with covariates**

. nl hockey anx0 disomean age income empl2 empl3 empl4 ed2 ed3 mar2 mar3 mar4 if blvswh ==1

(obs = 37)

Iteration 0: residual SS = 36095.08

Iteration 10: residual SS = 62.14749

Source | SS df MS Number of obs = 37

-------------+------------------------------ F( 13, 23) = 1.98

Model | 69.5822827 13 5.35248328 Prob > F = 0.0735

Residual | 62.147447 23 2.70206291 R-squared = 0.5282

-------------+------------------------------ Adj R-squared = 0.2616

Total | 131.72973 36 3.65915916 Root MSE = 1.643795

Res. dev. = 124.1893

(hockey)

------------------------------------------------------------------------------

anx0 | Coef. Std. Err. t P>|t| [95% Conf. Interval]

-------------+----------------------------------------------------------------

breakpoint | -.1937363 .0871276 -2.22 0.036 -.3739734 -.0134991

slope_l | -1.443646 2.061372 -0.70 0.491 -5.707919 2.820628

slope_r |  1.105118 .6127522 1.80 0.084 -.1624569 2.372692

age | .0279188 .1024385 0.27 0.788 -.1839915 .239829

income | .0669745 .0465939 1.44 0.164 -.0294124 .1633614

empl2 | .7045869 1.432913 0.49 0.628 -2.259619 3.668793

empl3 | 1.991683 1.346029 1.48 0.153 -.7927899 4.776156

empl4 | -.5537703 .7160691 -0.77 0.447 -2.035072 .9275314

ed2 | -1.376424 .7132385 -1.93 0.066 -2.851871 .0990217

ed3 | .7471211 1.294739 0.58 0.570 -1.931251 3.425493

mar2 | -1.478699 1.796669 -0.82 0.419 -5.195392 2.237993

mar3 | -2.611955 1.742018 -1.50 0.147 -6.215595 .9916843

mar4 | -2.198156 1.891985 -1.16 0.257 -6.112025 1.715713

cons | 13.80733 3.017571 4.58 0.000 7.565005 20.04965

------------------------------------------------------------------------------

**9. Note**

One can compute by hand the significance of the difference between two betas ∆β = (β_2_ - β_1_), using their estimates and SEs from the output, following

Clogg, C. C., Petkova, E., & Haritou, A. (1995). Statistical methods for comparing regression coefficients between models. American Journal of Sociology, 100(5), 1261-1293. doi:10.1086/230638

As detailed online at: [Stackexchange](https://stats.stackexchange.com/questions/93540/testing-equality-of-coefficients-from-two-different-regressions): <https://stats.stackexchange.com/questions/93540/testing-equality-of-coefficients-from-two-different-regressions>

One can use the z test: Z = (β_2_ - β_1_) / (SQRT[SE (β_1_)^2^ + SE (β_2_)^2^]

**10.** [**Multiple-Group Mixture (Latent Class) Analysis**](file:///C:\Users\MDPI\AppData\Local\Temp\preg_2class_mixture_105_2gr-reg_all-covs.out) **with Similar Setup as Hockey Stick Regression**

INPUT:

usevariables = anx0 disomean age income

ed2 ed3 mar2 mar3 mar4 empl2 empl3 empl4;

CLASSES = BlWh (2) Lupdown (2) ;! 2nd MvsL (2)is observed 'class'

KNOWNCLASS = BlWh (blvswh = 0 blvswh = 1);

ANALYSIS: TYPE = MIXTURE;

Model:

%overall%

anx0 on disomean age income ed2 ed3 mar2 mar3 mar4 empl2 empl3 empl4 ;

!~~~~~~~~~~~~~~~~~~two sections 1 for Observed classes!!!!!!!!!

!Model BlWh:

%BlWh#1.Lupdown#1% !Model white:

anx0 on disomean (WL1beta)

age income ed2 ed3 mar2 mar3 mar4 empl2 empl3 empl4 ;

disomean (WL1AveX);

%BlWh#1.Lupdown#2% !Model white:

anx0 on disomean (WL2beta)

age income ed2 ed3 mar2 mar3 mar4 empl2 empl3 empl4 ;

disomean (WL2AveX);

%BlWh#2.Lupdown#1% !Model white:

anx0 on disomean (BL1beta)

age income ed2 ed3 mar2 mar3 mar4 empl2 empl3 empl4 ;

disomean (BL1AveX);

%BlWh#2.Lupdown#2% !Model white:

anx0 on disomean (BL2beta)

age income ed2 ed3 mar2 mar3 mar4 empl2 empl3 empl4 ;

disomean (BL2AveX);

Model constraint:

NEW( dL1bBvsW dL2bBvsW);!DfqInt

dL1bBvsW = BL1beta-WL1beta; !will test within latent classes 1

dL2bBvsW = BL2beta-WL2beta; !will test within latent classes 1

OUTPUT: SAMPSTAT RESIDUAL tech1 tech4 CInterval stand MODINDICES ;

OUTPUT:

MODEL RESULTS USE THE LATENT CLASS VARIABLE ORDER

BLWH LUPDOWN

Latent Class Variable Patterns

BLWH LUPDOWN

Class Class

1 1

1 2

2 1

2 2

FINAL CLASS COUNTS AND PROPORTIONS FOR THE LATENT CLASS PATTERNS BASED ON THEIR MOST LIKELY LATENT CLASS PATTERN

Class Counts and Proportions

Latent Class

Pattern

1 1 15 0.21429

1 2 18 0.25714

2 1 18 0.25714

2 2 19 0.27143

CLASSIFICATION QUALITY

Entropy 0.956

Categorical Latent Variables

Means

BLWH#1 -0.114 0.239 -0.478 0.633

LUPDOWN#1 -0.071 0.250 -0.284 0.776

New/Additional Parameters

DL1BBVSW -1.262 0.198 -6.382 0.000 !difference in betas ∆β(Black-white) in latent class 1

DL2BBVSW 6.244 0.265 23.549 0.000 !difference in betas ∆β(Black-white) in latent class 2

| Estimate S.E. Est./S.E. P-Value  Latent Class Pattern 1 1 ! White women Latent CLass 2 n = 15  ANX0 ON  DISOMEAN 2.410 0.071 33.820 0.000  AGE 0.523 0.017 30.168 0.000  INCOME 0.211 0.006 32.496 0.000  ED2 -1.737 0.126 -13.811 0.000  ED3 -6.187 0.104 -59.662 0.000  MAR2 3.132 0.175 17.882 0.000  MAR3 5.232 0.124 42.119 0.000  MAR4 2.046 0.100 20.552 0.000  EMPL2 1.670 0.113 14.823 0.000  EMPL3 -2.064 0.109 -18.887 0.000  EMPL4 -0.105 0.101 -1.035 0.301  Means  DISOMEAN -0.248 0.173 -1.435 0.151  Intercepts  ANX0 1.932 0.500 3.865 0.000  Variances  DISOMEAN 0.414 0.134 3.098 0.002  Residual Variances  ANX0 0.121 0.028 4.242 0.000 | Estimate S.E. Est./S.E. P-Value  Latent Class Pattern 2 1 ! Black women Latent CLass 2 n = 18  ANX0 ON  DISOMEAN 1.147 0.185 6.210 0.000  AGE 0.050 0.055 0.899 0.369  INCOME 0.090 0.022 4.002 0.000  ED2 3.209 0.415 7.737 0.000  ED3 5.160 0.515 10.016 0.000  MAR2 0.636 0.231 2.752 0.006  MAR3 -2.741 0.489 -5.606 0.000  MAR4 -2.266 0.665 -3.407 0.001  EMPL2 -1.622 0.293 -5.545 0.000  EMPL3 5.918 0.183 32.360 0.000  EMPL4 -1.683 0.510 -3.298 0.001  Means  DISOMEAN -0.062 0.141 -0.436 0.663  Intercepts  ANX0 11.512 1.148 10.027 0.000  Variances  DISOMEAN 0.355 0.107 3.336 0.001  Residual Variances  ANX0 0.121 0.028 4.242 0.000 |
| --- | --- |
| Estimate S.E. Est./S.E. P-Value  Latent Class Pattern 1 2 ! White women Latent CLass 2 n = 18  ANX0 ON  DISOMEAN -5.740 0.129 -44.496 0.000  AGE -0.052 0.046 -1.132 0.258  INCOME 0.060 0.009 6.316 0.000  ED2 -1.009 0.191 -5.288 0.000  ED3 0.043 0.408 0.106 0.915  MAR2 -9.466 0.197 -47.934 0.000  MAR3 -9.272 0.201 -46.226 0.000  MAR4 -6.025 0.316 -19.053 0.000  EMPL2 -5.298 0.163 -32.554 0.000  EMPL3 -2.276 0.158 -14.385 0.000  EMPL4 -3.661 0.119 -30.652 0.000  Means  DISOMEAN -0.279 0.122 -2.292 0.022  Intercepts  ANX0 25.564 0.864 29.604 0.000  Variances  DISOMEAN 0.194 0.103 1.877 0.061  Residual Variances  ANX0 0.121 0.028 4.242 0.000 | Estimate S.E. Est./S.E. P-Value  Latent Class Pattern 2 2 ! Black women Latent CLass 2 n = 19  ANX0 ON  DISOMEAN 0.503 0.233 2.157 0.031  AGE 0.067 0.032 2.120 0.034  INCOME 0.030 0.026 1.151 0.250  ED2 -3.812 0.444 -8.586 0.000  ED3 -0.329 0.275 -1.196 0.232  MAR2 -0.927 0.452 -2.051 0.040  MAR3 -4.714 0.461 -10.227 0.000  MAR4 -4.686 0.536 -8.737 0.000  EMPL2 3.390 0.292 11.618 0.000  EMPL3 3.269 0.411 7.949 0.000  EMPL4 -0.770 0.284 -2.711 0.007  Means  DISOMEAN 0.613 0.149 4.126 0.000  Intercepts  ANX0 17.822 1.168 15.258 0.000  Variances  DISOMEAN 0.382 0.120 3.180 0.001  Residual Variances  ANX0 0.121 0.028 4.242 0.000 |
